# Bariatric Surgery Closure During COVID-19 Lockdown in Italy: The Perspective of Waiting List Candidates

**DOI:** 10.3389/fpubh.2020.582699

**Published:** 2020-11-17

**Authors:** Emanuela Bianciardi, Claudio Imperatori, Cinzia Niolu, Michela Campanelli, Marzia Franceschilli, Lorenzo Petagna, Francesca Zerbin, Alberto Siracusano, Paolo Gentileschi

**Affiliations:** ^1^Chair of Psychiatry, Department of Systems Medicine, University of Rome “Tor Vergata”, Rome, Italy; ^2^Cognitive and Clinical Psychology Laboratory, Department of Human Science, European University of Rome, Rome, Italy; ^3^Obesity Unit, Department of Surgery, University of Rome “Tor Vergata”, Rome, Italy

**Keywords:** mental health, depression, eating disorder, waiting list, obesity, COVID-19, bariatric Surgery

## Abstract

**Background:** From the beginning of March 2020, lockdown regimens prevented patients with obesity from receiving bariatric surgery. Surgical emergencies and oncological procedures were the only operations allowed in public hospitals. Consequently, patients with morbid obesity were put in a standby situation. With the aim at exploring the viewpoint of our future bariatric surgery patients, we built a questionnaire concerning obesity and COVID-19.

**Method:** A total of 116 bariatric surgery candidates were approached using a telephonic interview during the Italian lockdown.

**Results:** Of the total sample, 73.8% were favorable to regular bariatric surgery execution. Forty percent were concerned about their own health status due to the COVID-19 emergency, and 61.1% were troubled by the temporary closure of the bariatric unit. The majority of the sample were eating more. Forty-five percent and the 27.5% of patients reported a worsening of the emotional state and physical health, respectively. Most of the patients (52.2%) considered themselves more vulnerable to COVID-19, especially individuals with class III obesity. Patients who reported an increased consumption of food were younger (43.44 ± 12.16 vs. 49.18 ± 12.66; *F* = 4.28, *p* = 0.042). No gender difference emerged.

**Conclusion:** The lockdown had a negative result on Italian patients' psychological well-being and eating habits. The majority of patients would have proceeded with the surgery even during the COVID-19 emergency. Effective management and bariatric surgery should be restarted as soon as possible.

## Introduction

The pandemic of coronavirus disease 2019 (COVID-19) is challenging the world in unprecedented ways. Given the lack of herd immunity and effective vaccines or antiviral therapies, health systems are, everywhere, experiencing a tremendous strain and a significant mortality rate. Approximately 14% of COVID-19 patients develop serious disease requiring hospitalization and 5% need admission to intensive care units (1).

Obesity-related conditions seem to worsen the effect of coronavirus infection, and various reports have documented that people with heart disease and diabetes are at a higher risk of COVID-19 complications (2). This is particularly true in the younger population (3). In addition, the high rate of obesity around the world will probably cause a relevant percentage of humans to be at greater risk of contracting coronavirus and of dying from it.

Bariatric surgery is by far the most effective therapy for morbid obesity and metabolic syndrome (4–6). All over the world, from the beginning of March 2020, lockdown regimens prevented patients with obesity from receiving bariatric surgery (7). Surgical emergencies and oncological procedures were selected as the only surgical operations allowed in public hospitals. Consequently, patients with morbid obesity were put in a standby situation, which, in most cases, had a detrimental effect on their condition. In addition, in persons with obesity, self-isolation and the avoidance of social contact may trigger a high burden of psychological distress at a time when appointments with health care providers are limited (8–10). For these reasons, we believe that now, more than ever, health care providers need to fight obesity and its complications. The aim of this study was to determine the real impact of coronavirus pandemic on fears, desire, and expectations of bariatric surgery candidates to be treated as they were in a pre-COVID-19 era. Moreover, we explored if their perception of physical and mental health could have influenced this opinion. In order to explore the viewpoint of our future bariatric patients, we built a brief questionnaire concerning obesity and COVID-19 and submitted it to them.

## Materials and Methods

### Participants and Procedures

A total of 116 patients, referred to the Obesity Unit at the University of Rome “Tor Vergata” and waiting for bariatric surgery, entered the study. All patients were assessed during the Italian lockdown (between March 9 and May 4, 2020) by means of a telephone interview performed by a trained researcher. The telephonic interview was composed of socio-demographic items (e.g., employed before and during the lockdown, own accommodation during lockdown, etc.) and dichotomous (Yes/No) questions (Q) about physical and mental health in relation to the COVID-19 emergency (see Table 1). All the participants provided informed digital consent. The study was performed in accordance with the Helsinki declaration standards and was approved by the Institutional Ethics Review Committee of the University of Rome “Tor Vergata.” The study was registered and will be posted on the ClinicalTrials.gov public website with identifier: NCT04453579.

**Table 1:** Demographic and clinical data of patients.

**Variables**	**Total (*N*= 80)**	**Obesity Class I (*N*= 16)**	**Obesity Class II (*N*= 24)**	**Obesity Class III (*N*= 40)**	**Test**	***p***	***Post hoc***
Age, m ± SD	46.24 ± 12.66	44.44 ± 11.71	43.83 ± 13.56	48.00 ± 12.51	*F*_79_ = 0.85	0.432	–
Female, *N* (%)	52 (65.0)	13 (81.2)	15 (62.5)	24 (64)	χ^2^ = 2.36	0.31	–
Educational Level (years), M ± SD	11.51 ± 3.52	11.88 ± 3.22	11.54 ± 3.12	11.35 ± 3.87	*F*_79_ = 0.13	0.882	–
Employed before the lockdown, *N* (%)	48 (60.0)	10 (62.5)	13 (54.2)	25 (62.5)	χ^2^ = 0.49	0.784	–
Employed during the lockdown, *N* (%)	30 (37.5)	5 (31.2)	5 (20.8)	20 (50.0)	χ^2^ = 5.79	0.056	–
Own accommodation during lockdown, *N* (%)	64 (80.0)	12 (75.0)	18 (75.0)	34 (85.0)	χ^2^ = 1.25	0.535	–
Any Medical comorbidity, *N* (%)	37 (46.3)	6 (37.5)	6 (25.0)	25 (62.5)	χ^2^ = 9.10	**0.011**	III > II
Any Psychiatric disorders, *N* (%)	9 (11.3)	1 (6.2)	3 (12.5)	5 (12.5)	χ^2^ = 0.50	0.778	–
BMI, M ± SD	42.40 ± 8.92	32.84 ± 1.40	37.49 ± 1.50	49.16 ± 7.74	*F*_79_ = 61.51	**<0.001**	III > II; III > I; II > I
Q1 Agreement with the close of bariatric unit, *N* (%)	49 (61.3)	5 (31.2)	18 (75.0)	26 (65.0)	χ^2^ = 8.22	**0.016**	I > II
Q2 Concern about own health due to COVID-19 emergency, *N* (%)	32 (40.0)	4 (25.0)	10 (41.7)	18 (40.0)	χ^2^ = 1.94	0.378	–
Q3 Emotional state worsening due to COVID-19, *N* (%)	36 (45.0)	6 (37.5)	12 (50.0)	18 (45.0)	χ^2^ = 0.61	0.739	–
Q4 Physical state worsening due to the lockdown, *N* (%)	22 (27.5)	6 (37.5)	7 (29.2)	9 (22.5)	χ^2^ = 1.34	0.512	–
Q5 Worsening of medical comorbidities during the lockdown, *N* (%)	5 (6.3)	1 (6.2)	0 (0.0)	4 (10.0)	χ^2^ = 2.56	0.278	–
Q6 Treatment changes during the lockdown, *N* (%)	1 (1.3)	0 (0.0)	1 (4.2)	0 (0.0)	χ^2^ = 2.36	0.307	–
Q7 Concern about own weight due to ambulatory control stop, *N* (%)	39 (48.8)	37.5 (6)	12 (50)	21 (52.5)	χ^2^ = 1.05	0.591	–
Q8 Agreement with bariatric surgery during COVID-19 emergency, *N* (%)	59 (73.8)	12 (75.0)	16 (66.7)	31 (77.5)	χ^2^ = 0.93	0.630	-
Q9 More hungry during COVID-19 emergency, *N* (%)	24 (30.0)	5 (31.2)	6 (25.0)	13 (32.5)	χ^2^ = 0.42	0.812	–
Q10 Eat more during COVID-19 emergency, *N* (%)	41 (51.3)	11 (68.8)	10 (41.7)	20 (50)	χ^2^ = 2.87	0.238	–
Q11 Feeling sad due to the stop of bariatric surgery, *N* (%)	49 (61.0)	8 (50.0)	13 (54.2)	28 (70.0)	χ^2^ = 2.65	0.266	–
Q12 Concern to be more at risk to COVID-19 due to own obesity, *N* (%)	42 (52.2)	6 (37.5)	8 (33.3)	40 (100.0)	χ^2^ = 9.89	**0.007**	III > II; III > I

*BMI, body mass index; Q, question; COVID-19, Coronavirus Disease 19; Data in bold indicates statistically significant values*.

### Statistical Analyses

All analyses were performed with SPSS 18.0 statistical package for the social sciences (IBM, Armonk, NY, USA). Descriptive statistics have been calculated for the entire sample. Furthermore, one-way analysis of variance (ANOVA) analyses for continuous variables and chi-square analyses for categorical variables were used to examine group differences among (i) patients with class I obesity, (ii) patients with class II obesity, and (iii) patients with class III obesity. *Post hoc* tests (i.e., Tamhane T2 and single chi-square) were used when needed. Differences between groups were also explored considering (1) gender difference, (2) patients employed or unemployed during lockdown, and (3) patients who modified their diet (i.e., ate more) during lockdown. All the tests were considered significant for *p* < 0.05.

## Results

Of the 116 bariatric surgery candidates that were on the Obesity Unit waiting list, a total of 80 patients (52 women and 28 men; mean age: 44.24 ± 12.66) answered the questionnaire with a participation rate of 69%. The remaining 36 patients did not participate in the study for various reasons: unable to be contacted (14), unwilling to answer (3), already submitted to surgery in other institutions (7), and declined surgery (12).

The median waiting time for bariatric surgery of enrolled candidates was 147 days (range: 6–660). In 2019, the median waiting time for the operation was about 180 days. Patients had an average BMI of 42.40 kg/m^2^ (SD = 8.92). According to the standard BMI cutoff (11), there were 16 class I (20%), 24 class II (30.0%), and 40 class III (40%) patients with obesity (Table 1). None of the patients was found positive for COVID-19 and only two reported personal contacts with positive COVID-19 individuals.

During the lockdown, the majority of the study participants (80.0%) were living in personal homes and were unemployed (62.5%). Despite the majority of the sample (61.3%) being in agreement with the temporary closure of the bariatric unit due to the COVID-19 emergency (Q1), 59 patients (73.8%) would have proceeded with the surgery even during the COVID-19 emergency.

Thirty-two patients (40%) were concerned about their own health status due to COVID-19 emergency (Q2), and 61.1% of the sample were upset by the temporary closure of the bariatric unit (Q11). Furthermore, due to the temporary closure of the bariatric unit, 48.8% of the sample reported worries about the status of their own weight (Q10).

The majority of the sample (51.3%) also reported eating more during the COVID-19 emergency (Q10). Furthermore, in relation to the COVID-19 emergency, thirty-six (45%) and twenty-two (27.5%) patients reported a worsening of their emotional state (Q3) and physical health (Q4), respectively. The majority of the patients (52.2%) also considered themselves more vulnerable to COVID-19 because of their weight (Q12), especially individuals with class III obesity (100%) compared to both class I (37.5%) and class II (33.3) patients (χ^2^ = 9.89, *p* = 0.007).

With regard to gender differences, no statistical differences were observed for either socio-demographic variables or any of the Qs related to COVID-19 (Supplementary Table 1). No statistical differences were observed between employed and unemployed patients during the lockdown with the exception of Q5 (Supplementary Table 2): compared to unemployed patients, those who were employed during the lockdown reported a worsening of medical comorbidities (13.3 vs. 2.0%; χ^2^ = 4.11, *p* = 0.043).

Finally, several differences have been observed when considering patients who modified their diet (i.e., eating more) during the lockdown (Table 2). Patients who reported an increase in food consumption during the lockdown were (i) younger (43.44 ± 12.16 vs. 49.18 ± 12.66; *F* = 4.28, *p* = 0.042), (ii) in agreement with the performance of bariatric surgery during the COVID-19 emergency (87.8 vs. 59.0%; χ^2^ = 8.58, *p* = 0.003), (iv) in disagreement with the closure of the bariatric unit (53.7 vs. 23.1%; χ^2^ = 7.88, *p* = 0.005), (v) preoccupied about the interruption to bariatric surgery procedures (73.2 vs. 48.7%; χ^2^ = 5.04, *p* = 0.025). Furthermore, these patients also reported a worsening of both their emotional (56.1 vs. 33.3%; χ^2^ = 4.19, *p* = 0.041) and physical (41.5 vs. 12.8; χ^2^ = 8.23, *p* = 0.004) state due to the COVID-19 emergency.

**Table 2:** Differences between patients who modified their eating habits.

**Variables**	**Eat less during less COVID-19 emergency (*N*= 39)**	**Eat more during COVID-19 emergency (*N*= 41)**	**Test**	***p***
Age, m ± SD	49.18 ± 12.66	43.44 ± 12.16	*F*_79_ = 4.28	**0.042**
Women, *N* (%)	24 (61.5)	28 (68.3)	χ^2^ = 0.40	0.527
Educational Level (years), M ± SD	11.49 ± 3.80	11.54 ± 3.27	*F*_79_ = 0.01	0.950
Employed before the lockdown, *N* (%)	21 (53.8)	27 (65.9)	χ^2^ = 1.20	0.273
Employed during the lockdown, *N* (%)	16 (41.0)	14 (34.1)	χ^2^ = 0.40	0.525
Own accommodation during lockdown, *N* (%)	31 (79.5)	80.5 (33)	χ^2^ = 0.01	0.911
Any Medical comorbidity, *N* (%)	17 (43.6)	20 (48.8)	χ^2^ = 0.22	0.642
Any Psychiatric disorders, *N* (%)	2 (5.1)	7 (17.1)	χ^2^ = 2.86	0.091
BMI, M ± SD	43.72 ± 9.79	41.13 ± 7.93	*F*_79_ = 1.70	0.196
Q1 Agreement with the close of bariatric unit, *N* (%)	30 (76.9)	19 (46.3)	χ^2^ = 7.88	**0.005**
Q2 Concern about own health due to COVID-19 emergency, *N* (%)	12 (30.8)	20 (48.8)	χ^2^ = 2.70	0.100
Q3 Emotional state worsening due to COVID-19, *N* (%)	13 (33.3)	23 (56.1)	χ^2^ = 4.19	**0.041**
Q4 Physical state worsening due to the lockdown, *N* (%)	5 (12.8)	17 (41.5)	χ^2^ = 8.23	**0.004**
Q5 Worsening of medical comorbidities during the lockdown, *N* (%)	1 (2.6)	4 (9.8)	χ^2^ = 1.76	0.184
Q6 Treatment changes during the lockdown, *N* (%)	0 (0.0)	1 (2.4)	χ^2^ = 2.4	0.963
Q7 Concern about own weight due to ambulatory control stop, *N* (%)	15 (38.5)	24 (58.5)	χ^2^ = 3.22	0.073
Q8 Agreement with bariatric surgery during COVID-19 emergency, *N* (%)	23 (59.0)	36 (87.8)	χ^2^ = 8.58	**0.003**
Q9 More hungry during COVID-19 emergency, *N* (%)	4 (10.3)	20 (48.8)	χ^2^ = 14.13	**<0.001**
Q11 Feeling sad due to the stop of bariatric surgery, *N* (%)	19 (48.7)	30 (73.2)	χ^2^ = 5.04	**0.025**
Q12 Concern to be more at risk to COVID-19 due to own obesity, *N* (%)	19 (48.7)	23 (56.1)	χ^2^ = 0.44	0.509

*BMI, body mass index; Q, question; COVID-19, Coronavirus Disease 19; Data in bold indicates statistically significant values*.

## Discussion

The duo “obesity-COVID-19” is and most likely will be a strong determining factor of mortality in countries where both coronavirus infection and obesity frequently affect the population. It is widely reported and accepted by most researchers that patients with morbid obesity, type II diabetes, hypertension, and metabolic syndrome are at higher risk of mortality when infected by coronavirus (6). Other consequences of these two diseases need attention. Patients with obesity who become ill and infected with coronavirus will probably require intensive care monitoring and treatment. Having an underlying pulmonary dysfunction and being difficult to intubate make management of these individuals challenging. Furthermore, weight and size limit restrictions on imaging machines will make it harder to obtain diagnostic imaging. Also, these patients may not do well when prone, and nursing staff will find it difficult to position and transport them. Special beds and dedicated equipment are only available in specialized units and not widely present, elsewhere in hospitals, certainly not in every country. Many health systems are not well enough set up to manage patients with obesity and the current crisis will only underline these limitations even more.

This global pandemic is quickly becoming a serious economic crisis, which will affect the world's most vulnerable population, those who are at higher risk of obesity. Consequently, it might contribute to an increase in obesity rates since weight loss interventions (such as surgery) are being severely curtailed at present. Lockdown measures, as introduced in many countries, will have an impact on mobility and enforced physical inactivity, resulting in an increased risk of metabolic complications. In addition, the need for self-isolation is prompting many individuals to rely on processed and canned food, with a longer shelf life, as an alternative to fresh produce, resulting in an increase in weight over an extended period.

During the COVID-19 outbreak, bariatric surgery has been postponed in all countries, where it was frequently performed, due to the need to free up inpatient capacity and the possibility of viral contagion in high-risk patients with obesity (12). With the return to normal services, weight loss surgery should be reintroduced in bariatric centers everywhere. To our knowledge, no studies, during the last months, have investigated the evolution of both physical and mental conditions in these patients.

The rationale of this study was, therefore, to perform an extensive analysis of patients' opinion on bariatric surgery closure through the administration of a questionnaire investigating various aspects of their life and clinical path during the COVID-19 outbreak. With the return to standard volumes of bariatric surgeries, a selection of patients will, probably, be needed in most centers. Various authors and National Obesity Surgery Societies have already reported different strategies to reintroduce bariatric surgery while mitigating the harm caused by post-operative complications in these patients (6). For this reason, we believe that a clear picture of our patients was needed in order to highlight risky practices and clinical factors, thereby prompting us to generate an effective selection of surgical patients (13, 14). We were also interested in studying the effects of isolation and smart working on such fragile individuals after bariatric surgery, the only effective therapy for their condition, had been stopped.

A study investigating the impact of COVID-19 on patients included in a bariatric surgery program (i.e., waiting for bariatric surgery or attending a post-bariatric follow-up) showed that those individuals perceived an increased level of psychological distress along with a worsening of the eating behaviors (15). Accordingly, the majority of our sample reported eating more during the COVID-19 emergency. In addition, we specifically asked patients if the COVID-19 lockdown had an impact on their mental health and eating habits. As regards the COVID-19 emergency, 45 and 27.5% of patients reported a worsening of their emotional state and physical health, respectively. Forty percent of them were concerned about their own health status due to COVID-19 emergency, and 61.1% of the sample were upset about the temporary closure of the bariatric unit. Furthermore, due to the temporary closure of the bariatric unit, 41.2% of the sample reported concerns about their own clinical status and 48.8% were worried about their weight. Despite the majority of the sample being in agreement with the temporary closure of the bariatric unit due to the COVID outbreak, 73.8% of patients favored proceeding with the surgery even during the COVID-19 emergency. This is the first important outcome of our study. Patients perceive morbid obesity as more dangerous than coronavirus infection and they are well informed about the risk of morbidity and mortality for their disease. Due to their weight, especially those with class III obesity, most of the patients also consider themselves more vulnerable to COVID-19. This phenomenon is clearly demonstrated in the scientific literature. Patients with obesity and metabolic syndrome are at greater risk of COVID-19 complications, which can be harmful even in young people (16). In this setting, various mechanisms have been suggested to increase the risk from viral infections, including reduced natural killer cell number and activity, impaired antigen-stimulation responses and low-grade chronic inflammation (17).

As expected, but clearly shown in our survey, isolation had a negative effect on patients' psychological well-being and on their eating habits. Several differences have been observed when considering patients who modified their diet (i.e., eating more) during the lockdown. Patients who reported an increase in food consumption during the lockdown were younger, in agreement with the performance of bariatric surgery, even during COVID-19 emergency, in disagreement with the closure of bariatric unit, and upset by the cessation of bariatric surgery. Furthermore, these patients also reported a worsening of both emotional and physical conditions due to COVID-19 emergency. These results are, in our opinion, a further demonstration that bariatric surgery is essential for the cure of morbid obesity and should be restarted as soon as possible. With the escalation of government restrictions came an unforeseen change in the Italian epidemiological scenario. Moreover, Italian clinicians were on the frontline of the corona war. All these reasons contributed to the fact that in many bariatric centers, it was not possible to provide a prompt multidisciplinary health care system as it was the case of our hospital that was selected as a COVID hospital from the government. Thus, at the time of the telephonic interview, patients were not supported by any telemedicine service. The COVID-19 emergency saw, in only 2 months, our patients experience a worsening of their emotional state and physical health. They considered themselves to be more vulnerable to coronavirus infection, because of their weight, especially individuals with a more severe degree of obesity. No previous global circumstances, in only 2 months, delivered such a terrible impact on the social and clinical life of the population suffering from obesity. Isolation, smart working, worsening of eating habits due to isolation, absence of physical exercise, and closure of outpatient services are all factors that may have contributed to this phenomenon. Moreover, the closure of bariatric surgery will prolong the median waiting time for bariatric surgery compared to the year 2019. In addition, social distancing and the need for isolation determined the interruption of all outpatient services dedicated to bariatric patients. For these reasons, we expect an increase in mortality rates in patients with obesity, in the near future. Effective management and bariatric surgery should be restarted as soon as possible. We recognize some limitation of the study. First of all, patients were not submitted to validated self-report questionnaires for the assessment of eating disorder and depression. Even though our main aim was to explore patients' opinion about their mental health status, we searched for the association with the attitude to bariatric surgery closure. Moreover, we could not compare our results to the pre-lockdown psychological status because many patients have not yet been submitted to the psychosocial behavioral assessment by our staff. Finally, we could have compared our data to a sample of parous individuals with obesity that were not seeking for surgery.

Above these limitations, we believe in the significance of our results. To date, this is the first report exploring the viewpoint of individuals with severe obesity that are waiting for surgery. We highlighted that those suffering from a more severe degree of obesity were worried about their health status and were in disagreement with the closure of surgery. As bariatric surgery may be considered the last best hope (18), the closure of waiting list may deprive such individuals of the hope of a better future. Now that the possibility of a second wave of COVID-19 infections was predicted (19), our findings may improve the planning of bariatric surgery waiting list according to the burden of comorbidities. Although severe obesity is a chronic progressive condition, the increase of waiting time for its treatment may worsen patients' prognosis and suffering. Accordingly, our data may also strengthen the rationale for classifying some bariatric surgery interventions as urgent rather than elective.

## Data Availability Statement

The raw data supporting the conclusions of this article will be made available by the authors, without undue reservation.

## Ethics Statement

All the participants provided informed digital consent. The study was performed in accordance with the Helsinki declaration standards and was approved by the Institutional Ethics Review Committee of the University of Rome Tor Vergata. The study was registered and will be posted on the ClinicalTrials.gov public website with identifier: NCT04453579.

## Author Contributions

EB and PG designed the study and developed the questionnaire and wrote the manuscript. LP, MF, FZ, and MC collected the data from the telephone interviews and completed the database. CI and PG performed the statistics. CI wrote the results of the manuscript and prepared tables. EB, PG, CN, AS, and CI revised the manuscript. All authors contributed to the article and approved the submitted version.

## Conflict of Interest

The authors declare that the research was conducted in the absence of any commercial or financial relationships that could be construed as a potential conflict of interest.
