# Supplementary material for: Bariatric Surgery Closure During COVID-19 Lockdown in Italy: The Perspective of Waiting List Candidates
Source: Front Public Health. 2020 Nov 17;8:582699. doi: 10.3389/fpubh.2020.582699 (PMC7706656; doi:10.3389/fpubh.2020.582699)
Supplement: Supplementary file 1 [file Table_1.DOCX]

**Supplementary Table 1.** Gender differences

| **Variables** | **Men**  **(N= 28)** | **Women**  **(N= 51)** | **Test** | ***p*** |
| --- | --- | --- | --- | --- |
| Age – M±SD | 47.25±12.45 | 45.69±12.86 | F_79_= 0.27 | 0.603 |
| Educational Level (years) - M±SD | 12.00±3.09 | 11.25±3.73 | F_79_= 0.83 | 0.366 |
| Employed before the lockdown – N (%) | 20 (71.4) | 28 (53.8) | χ^2^= 2.34 | 0.126 |
| Employed during the lockdown – N (%) | 13 (46.4) | 17 (32.7) | χ^2^= 1.47 | 0.226 |
| Own accommodation during lockdown – N (%) | 23 (82.1) | 41 (78.8) | χ^2^= 0.12 | 0.725 |
| Any Medical comorbidity – N (%) | 11 (39.3) | 26 (50.0) | χ^2^= 0.84 | 0.359 |
| Any Psychiatric disorders – N (%) | 3 (10.7) | 6 (11.5) | χ^2^= 0.01 | 0.911 |
| BMI – M±SD | 43.90±8.11 | 41.59±9.30 | F_79_= 1.23 | 0.272 |
| Q1 Agreement with the close of bariatric unit – N (%) | 21 (75.0) | 28 (53.8) | χ^2^= 3.43 | 0.064 |
| Q2 Concern about own health due to COVID-19 emergency – N (%) | 11 (39.3) | 21 (40.4) | χ^2^= 0.01 | 0.924 |
| Q3 Emotional state worsening due to COVID-19 – N (%) | 11 (39.3) | 25 (48.1) | χ^2^= 0.57 | 0.451 |
| Q4 Physical state worsening due to the lockdown – N (%) | 6 (21.4) | 16 (30.8) | χ^2^= 0.80 | 0.372 |
| Q5 Worsening of medical comorbidities during the lockdown – N (%) | 3 (10.7) | 2 (3.8) | χ^2^= 1.47 | 0.226 |
| Q6 Treatment changes during the lockdown – N (%) | 0 (0.0) | 1 (1.9) | χ^2^= 0.55 | 0.460 |
| Q7 Concern about own weight due to ambulatory control stop – N (%) | 12 (42.9) | 27 (51.9) | χ^2^= 0.60 | 0.439 |
| Q8 Agreement with bariatric surgery during COVID-19 emergency – N (%) | 21 (75.0) | 38 (73.1) | χ^2^= 0.04 | 0.852 |
| Q9 More hungry during COVID-19 emergency – N (%) | 13 (46.4) | 17 (32.7) | χ^2^= 1.47 | 0.226 |
| Q10 Eat more during COVID-19 emergency – N (%) | 13 (46.4) | 28 (53.8) | χ^2^= 0.40 | 0.527 |
| Q11 Eat less during COVID-19 emergency – N (%) | 2 (7.1) | 6 (11.5) | χ^2^= 0.39 | 0.532 |
| Q12 Concern to be more at risk to COVID-19 due to own obesity – N (%) | 11 (39.3) | 31 (59.6) | χ^2^= 3.02 | 0.082 |
| Abbreviation: BMI = body mass index; Q= question COVID-19 = Corona Virus Disease 19 | | | | |
