# Supplementary material for: Bariatric Surgery Closure During COVID-19 Lockdown in Italy: The Perspective of Waiting List Candidates
Source: Front Public Health. 2020 Nov 17;8:582699. doi: 10.3389/fpubh.2020.582699 (PMC7706656; doi:10.3389/fpubh.2020.582699)
Supplement: Supplementary file 2 [file Table_2.DOCX]

**Supplementary Table 2.** Differences between employed and unemployed patients during the lockdown

| **Variables** | Unemployed during the lockdown  **(N= 50)** | Employed during the lockdown  **(N= 30)** | **Test** | ***p*** |
| --- | --- | --- | --- | --- |
| Age – M±SD | 46.32±13.44 | 46.10±11.45 | F_79_= 0.01 | 0.941 |
| Women – N (%) | 35 (70) | 17 (56.7) | χ^2^= 1.47 | 0.226 |
| Educational Level (years) - M±SD | 11.18±3.35 | 12.07±3.76 | F_79_= 1.20 | 0.277 |
| Own accommodation during lockdown – N (%) | 37 (74.0) | 27 (90.0) | χ^2^= 3.00 | 0.083 |
| Any Medical comorbidity – N (%) | 21 (42.0) | 16 (53.3) | χ^2^= 0.97 | 0.325 |
| Any Psychiatric disorders – N (%) | 5 (10.0) | 4 (13.3) | χ^2^= 0.21 | 0.648 |
| BMI – M±SD | 41.47±8.50 | 43.93±9.53 | F_79_= 1.42 | 0.237 |
| Q1 Agreement with the close of bariatric unit – N (%) | 32 (64.0) | 17 (56.7) | χ^2^= 0.43 | 0.515 |
| Q2 Concern about own health due to COVID-19 emergency – N (%) | 20 (40.0) | 12 (40.0) | χ^2^= 0.00 | 1.000 |
| Q3 Emotional state worsening due to COVID-19 – N (%) | 26 (52.0) | 10 (33.3) | χ^2^= 2.64 | 0.104 |
| Q4 Physical state worsening due to the lockdown – N (%) | 15 (30.0) | 7 (23.3) | χ^2^= 0.42 | 0.518 |
| Q5 Worsening of medical comorbidities during the lockdown – N (%) | 1 (2.0) | 4 (13.3) | χ^2^= 4.11 | **0.043** |
| Q6 Treatment changes during the lockdown – N (%) | 1 (2.0) | 0 (0.0) | χ^2^= 0.61 | 0.436 |
| Q7 Concern about own weight due to ambulatory control stop – N (%) | 25 (50.0) | 14 (46.7) | χ^2^= 0.08 | 0.773 |
| Q8 Agreement with bariatric surgery during COVID-19 emergency – N (%) | 35 (70.0) | 24 (80.0) | χ^2^= 0.969 | 0.325 |
| Q9 More hungry during COVID-19 emergency – N (%) | 14 (28.0) | 10 (33.3) | χ^2^= 0.25 | 0.614 |
| Q10 Eat more during COVID-19 emergency – N (%) | 27 (54.0) | 14 (46.7) | χ^2^= 0.40 | 0.525 |
| Q11 Feeling sad due to the stop of bariatric surgery – N (%) | 31 (62.0) | 18 (60.0) | χ^2^= 0.03 | 0.859 |
| Q12 Concern to be more at risk to COVID-19 due to own obesity – N (%) | 29 (58.0) | 13 (43.3) | χ^2^= 1.62 | 0.203 |
| Abbreviation: BMI = body mass index; Q= question COVID-19 = Corona Virus Disease 19 | | | | |
